# Total Diet Replacement Within an Integrated Intensive Lifestyle Intervention for Remission of Type 2 Diabetes: Lessons From DiRECT

**DOI:** 10.3389/fendo.2022.888557

**Published:** 2022-06-02

**Authors:** Jarvis C. Noronha, George Thom, Michael E. J. Lean

**Affiliations:** ^1^ Toronto 3D (Diet, Digestive Tract and Disease) Knowledge Synthesis and Clinical Trials Unit, Clinical Nutrition and Risk Factor Modification Centre, St. Michael’s Hospital, Toronto, ON, Canada; ^2^ School of Medicine, Faculty of Medicine, The University of Queensland, Brisbane, QLD, Australia; ^3^ School of Medicine, Dentistry and Nursing, University of Glasgow, Glasgow, United Kingdom

**Keywords:** total diet replacement, obesity treatment, weight loss, diabetes, diabetes remission

## Abstract

The prognosis for people with type 2 diabetes (T2D) remains concerning, yet its seriousness is often underestimated. T2D is a manifestation, in susceptible individuals, of the disease-process of obesity, and at diagnosis, 10-year survival rates for T2D are around 50%. Here, we will examine: (a) the role of weight loss in T2D, (b) use of total diet replacements (TDRs) to induce weight loss, (c) the Diabetes Remission Clinical Trial (DiRECT) protocol and key results, (d) other dietary interventions related to T2D remission, (e) remission in real life, and (f) future directions. Remission of short-duration T2D will usually require 10-15% body weight loss, and results from the DiRECT trial demonstrated that this can be achieved within routine care in nearly half of all people undertaking a supported, TDR-led behavioural weight management programme. In light of these findings, which have since been replicated in the Diabetes Intervention Accentuating Diet and Enhancing Metabolism (DIADEM-I) trial conducted in the Middle East and North Africa, it is now time to prioritize weight loss programmes for T2D remission from diagnosis, and with increasing acceptance and availability of digital healthcare, there is an opportunity to scale up delivery of remission programmes in a cost effective manner.

## Introduction

The prognosis for people with T2D is not good, with 10-year survival rates around 50% (1), yet its seriousness is often underestimated. Surgical and non-surgical weight loss studies (2–5) have conclusively demonstrated that 10-15% body weight loss will lead to remission of type 2 diabetes (HbA1c<48 mmol/mol, without glucose-lowering medication for at least 3 months) in the majority of individuals with short duration disease. Remission is a relatively new concept, and prioritizing this as a principal target at diagnosis would represent a major shift in clinical care, where treatments have historically aimed to ‘control’ T2D.

It is well established that weight gain is the dominant cause of T2D among individuals who have an underlying predisposition to metabolic syndrome (6, 7). Many people can gain weight, even to high degrees of obesity, by depositing excess energy as fat into subcutaneous sites, without developing metabolic and macrovascular complications. However, a large subset of the population (probably predisposed by genetic and/or epigenetic factors) also start to deposit fat into ectopic sites and vital organs such as the heart, liver and pancreas. Excess fat damages organ functions, leading to development of hypertension, dyslipidemia, and T2D, and ultimately to multisystem metabolic failure and progressive vascular complications. These end-stage complications are not reversible. However, T2D should be reversible by treating the earlier underlying process of body fat accumulation.

## Role of Weight Loss in T2D

If weight gain is the dominant driver of T2D, then weight loss is the logical solution for management. This tenet is strongly supported by consistent evidence that relatively modest weight loss at an earlier stage, with pre-diabetes, can arrest the disease-process. Randomised trials in Finland and North America both reported 58% reductions in 4-year progression from prediabetes to T2D with weight loss of about 7-kg, maintained at 4-kg over 4 years (8, 9) subsequently repeated elsewhere (10).

Once T2D has developed, the clock starts ticking towards the arrival of permanent, painful and disabling microvascular complications, despite helpful guideline-directed medications. Bariatric surgery has demonstrated remissions to a non-diabetic state for at least 2 years in 70-80% of participants (11, 12), with weight losses ranging from 20-40%. However, bariatric surgery cannot be provided to all the vast numbers of people with T2D, and many are not interested in a surgical procedure for weight loss (13). Also, many people with T2D, particularly with Asian heritage, have BMI too low to warrant bariatric surgery.

The question, arises - how much weight loss is needed to achieve remission of T2D? The likelihood appears to be with weight loss above 10-kg/% of total body weight, for most people. A well-conducted randomized controlled trial of bariatric surgery found that 15% (~15-kg) weight loss resulted in remissions for nearly all participants (2). A T2D clinic audit from Scotland, where all were seen by dietitians at each visit, and were followed from diagnosis to death, found that for the average T2D patient, each 1-kg weight loss in the first year was associated with 3-4 months longer survival (14). Reliable data were available up to 14-kg weight loss, at which point survival was similar to the general population. A recent study of 5,928 patients with T2D who underwent bariatric surgery found that T2D remission was more likely with each 5% increase in total weight loss until 20% total weight loss, where remission rates did not increase substantially (3).

## Total Diet Replacements to Induce Weight Loss in T2D

Can weight loss of >10-kg/% of total body weight, if that is required for diabetes remission, be reliably achieved without bariatric surgery? One option, already widely available and used by the general population, is to use low-calorie total diet replacements (TDRs).

In a proof-of-concept study, eleven T2D participants consumed a 600 kcal/d liquid formula diet, supplemented with non-starchy vegetables, for 8 weeks. An age-, sex- and weight-matched group of 8 non-diabetic participants was also studied. Average weight loss was ~15-kg or ~15% of initial body weight in the seven T2D participants who completed the 8 weeks’ diet. This led to a return of normal beta cell function and hepatic glucose output, in association with marked decreases in pancreatic and liver fat (15).

A second study was conducted to assess the feasibility of achieving >15-kg weight loss over a longer duration (12 months) through a weight loss programme in routine primary care. Participants consumed a micronutrient-replete low-energy liquid diet (810–833 kcal/day) for 12 weeks or 20-kg weight loss (whichever occurred sooner), followed by structured food reintroduction (FR; 6-8 weeks) and continued support for weight loss maintenance. Of 91 participants who entered the programme, 30 (33%) had maintained weight loss of ≥15-kg at 12 months. Most preferred the commercial formula diet product, rather than having to make decisions about foods and control portion sizes themselves. Few elected to use optional orlistat (16).

## The Diabetes Remission Clinical Trial (DiRECT)

The Diabetes Remission Clinical Trial (DiRECT) was designed to investigate whether a weight management program delivered within routine primary care, thus potentially accessible to most people with T2D, could achieve the necessary weight loss to sustain remissions of T2D cost-effectively. Trial and baseline participant characteristics are outlined in **Table 1**, and intervention components in **Table 2**. All participants continued to receive routine diabetes care under current clinical guidelines.

**Table 1:** DiRECT characteristics and baseline participant characteristics.

TRIAL CHARACTERISTICS		
Design	Open-label, cluster-randomised trial	
Location	Primary care practices in Scotland and the Tyneside region of England	
Treatments Intervention group Control group	23 practices 26 practices	
Study duration	2 years	
**PARTICIPANT CHARACTERISTICS AT BASELINE**		
	Intervention group (N=149)	Control group (N=149)
Sex Male Female	83 (56%) 56 (38%)	66 (44%) 93 (62%)
Age (years)	52.9 (7.6)	55.9 (7.3)
BMI (kg/m^2^)	35.1 (4.5)	34.2 (4.3)
HbA1c (%)	7.7 (1.25)	7.5 (1.05)
Time since diabetes diagnosis (years)	3.0 (1.7)	7.5 (1.05)

Data are mean (SD) and adapted from (4).

**Table 2:** Intervention characteristics in DiRECT.

**Counterweight Plus Program**	Weeks 0 to 12: total diet replacement (TDR)
	Weeks 13 to 18: food re-introduction (FR)
	Weeks 19 to 104: weight maintenance (WM)
**Delivery of intervention**	The intervention was delivered to each patient individually, at their usual primary care center, by either a practice nurse or a local dietitian (if one was available). These practitioners received two days of training, and subsequent on-job mentoring from experienced specialist dietitians.
**Handling of medications**	As a key feature of the intervention, participants were asked to stop all oral hypoglycemic agents, antihypertensive and diuretic drugs upon commencement of TDR. This served two functions: first, as a safety measure to avoid potential hypoglycemia and postural hypotension associated with weight loss, and secondly as a strong incentive to adhere to the intervention. These medications were reintroduced (as per study protocol based on national clinical guidelines) if blood glucose or blood pressure rose above treatment thresholds.
**TDR Phase (Weeks 0 to 12)**	The TDR phase consisted of a micronutrient replete 825–853 kcal/d liquid formula diet (soups and shakes) provided by Cambridge Weight Plan (which owned Counterweight Ltd at that time) to replace usual foods, with ample fluids (2.25 L/day), for 12 weeks
**FR Phase (Weeks 13 to 19)**	The FR phase involved a stepped transition to a food-based WM diet.
**WM Phase (Weeks 19 to 104)**	During the WM phase, participants were advised to follow a food-based diet based on the “Eatwell” guidelines (15) with an individually tailored energy prescription (to support weight stabilization and prevent weight regain) with the option of using one sachet of formula diet per day. The macronutrient content of the maintenance diet was not formally prescribed, but the Eatwell guidelines target a reduction in total and long-chain saturated fatty acids, with approximately 50% of energy from carbohydrate.
**‘Rescue plan’**	Recognizing the potential for weight regain, the program included a firm commitment to provide relapse management. Correcting even small weight regains can be difficult, so participants were offered a further period of partial or full TDR if they regained >2 kg (partial TDR) or 4 kg (full TDR)

## Key Results from the Direct Trial

### Weight Loss

Participants lost a mean 14.5-kg during the TDR phase, with an average weight loss of 10-kg at year 1. Over the 2-year follow-up, regain of weight from the end of TDR was 7-kg, with an overall mean weight loss of 7.6kg from baseline (17).

### Diabetes Remission

Diabetes remission was defined, anticipating the international consensus agreement (18), as HbA1c<48 mmol/mol (<6.5%) without glucose-lowering medication for at least 3 months (to allow HbA1c to settle). DiRECT was powered to detect remissions in 1 in 5 randomised participants at 12 months, considered a clinically important result, and 5% remissions were expected for the controls. At 12 months, the intervention group remission rate of 46% greatly exceeded (by ~2-fold) the 20% considered important for re-defining national diabetes management strategy, versus 4% remission among controls. At 24 months, the intervention group remission rate was still 36% (4, 17).

### Diabetes Remission and Weight Change

The decline in remission from 46% to 36% between 12 to 24 months represents relapses for 15 participants whose weight regained to within 10kg of baseline. Intervention group participants who maintained remissions at 12 and 24 months (48/149 participants) lost 15.5-kg and 11.4-kg from baseline, respectively. Participants whose T2D relapsed between 12 and 24 months lost 12-kg at 12 months but only 4.9-kg at 24 months. Participants failing to achieve remission at either 12 or 24-months lost 5.8-kg at 12 and 5.6-kg at 24 months, respectively (4, 17).

Remissions were well sustained for a given weight change. Intervention group participants who achieved weight loss of >15 kg had remission rates at 12 and 24 months of 86% and 82% (19). With >10 kg weight loss, remission rates were above 75% (19). An extensive analysis of predictors of remission (19) revealed only minor effects among the baseline variables. There was slightly greater likelihood of remission for men than women, for those with lower HbA1c and on fewer medications. During the intervention there was no overall change in physical activity, as measured by accelerometers, despite encouragement and provision of step-counters (20). Collectively, these data underscore the importance of weight loss achieved through energy restriction (rather than energy expenditure) as the dominant factor for achieving and sustaining diabetes remission.

### Blood Pressure and Antihypertensive Needs

In the intervention group as a whole, mean systolic blood pressure (SBP) and diastolic blood pressure (DBP) fell by 10-mmHg and 2-mmHg, respectively, during TDR (up to 20-wks) (21). For those participants without hypertension, the falls in BP were immediate and quite substantial. In those previously treated for hypertension, who discontinued antihypertensive medications, there were no statistically significant changes in BP until visit 6 (~week 9), when SBP and DBP fell 4.5- and 2.5-mmHg, respectively. Falls in BP were slower for those who stopped two or more antihypertensives. In two-thirds of the participants who discontinued antihypertensive medications, some antihypertensive medications had to be recommenced using the DiRECT reintroduction protocol, but 33.3% were able to remain off all the discontinued antihypertensive and diuretic medications through to the end of TDR (21). At 24 months, mean SBP had decreased by 4.3-mmHg in the intervention group and 1.4-mmHg in the control group from baseline with more participants in the control group receiving antihypertensive medications (60% in control group vs. 47% in intervention group) (17). These results demonstrate the combined, potent hypotensive effects of negative energy balance, active weight loss and reduced sodium intake, as well as the (lesser) persistent hypotensive effect of sustained weight loss. The protocol used in DiRECT, to withdraw medications on day-one, and reintroduce them if and when blood pressure rose to treatment thresholds, proved safe.

### Other Secondary Outcomes at 24 Months

In the intervention group, mean HbA1c fell by 5.2 mmol/mol (0.5%) between baseline and 24 months with fewer participants requiring glucose-lowering medications (74% at baseline vs. 40% at 24 months). In the control group, there was no change in HbA1c with more participants receiving medications by 24 months (77% at baseline vs. 84% at 24 months) (17). Quality of life (assessed by visual analogue scale) at 24 months improved significantly more in the intervention than control group (17).

### Adverse Events and Drop-Out

The number of participants who withdrew within the first year were 26 (18%) (4). A further 16 (11%) withdrew during year-2 (17) so in total 29% withdrew prematurely from the trial. These individuals were still included in the intention to treat analyses, using data collected routinely in primary care. Information on minor adverse events expected during TDR (constipation, headache, dizziness, increased sensitivity to cold) were sought at each appointment, and dealt with by the practitioner. Most were mild and none resulted in drop out (4, 17).

Serious adverse events were no different between the treatment groups in year 1, but in year 2 there were significantly more in the control group, many obesity- and diabetes-related (4, 17).

### Economics

A detailed health economics analysis found that the cost of providing the Counterweight-Plus/DiRECT intervention in routine medical practice (including staff training etc.) was significantly offset by savings to National Health Service (NHS) care costs, through reductions of medication prescriptions, consultations and referrals, and serious adverse events. A lifetime projection estimated that people with T2D given the intervention would live longer, feel better (improved Quality of Life) and incur smaller medical care costs (22).

### Participant and Healthcare Practitioner Experience

Qualitative examinations have been conducted to understand the experiences of both participants and general practitioners (GPs) with the DiRECT intervention (23, 24). Overall, weight loss with TDR was easier than expected by both participants and GPs. Transition to regular food and weight maintenance was challenging and required more support. GPs were initially sceptical, and worried by stopping medications, but soon gained confidence after seeing results. GPs found DiRECT implementation easy, with only modest efforts needed to keep participants engaged, and all were extremely satisfied with participating. Many participating healthcare practitioners have since modified their usual practices in diabetes care, and place greater emphasis on weight loss and lifestyle changes from the outset (23, 24).

## Summary of Dietary Interventions Reporting T2D Remissions

A recent systematic review (25) found only two randomised controlled trials (DIADEM-I and DiRECT), of non-surgical weight management to examine remissions of T2D. The DIADEM-I trial was conducted to assess the generalizability of the DiRECT results, using a near-identical intervention protocol, to people with early T2D from the Middle East and north Africa. At 12 months, diabetes remission occurred in 61% of intervention participants (mean 12-kg weight loss) compared with 12% in the control group (mean 4-kg weight loss). The greater 12-month remission rates in DIADEM-I (61%) than DiRECT (46%) probably reflected shorter duration of diabetes (<2 years) and slightly greater weight loss, with more remissions in the control group also (12% in DIADEM-I vs. 4% in DiRECT) (5).

T2D remissions have also been reported with low-carbohydrate (ketogenic) diets, but in an open-label, non-randomised study only 19% achieved remission at 12 months, despite an average weight loss of 13.8-kg over 12-months. The protocol did not include withdrawal of glucose-lowering medication, so a higher figure is likely, but recent review evidence suggests no special advantage for low carbohydrate diets in diabetes care (25, 26).

In a remarkable English GP practice, patients with newly diagnosed and pre-existing T2D were offered advice on a low carbohydrate diet. An audit between 2013 and 2019 reported that of 473 patients with T2D, 27% opted to follow the low carbohydrate diet for a mean duration of 23 months T2D remission was achieved by 12.5% of patients, using intention-to-treat analysis (and 46% of ‘completers’ who adhered to the diet), with significant improvements in weight, blood pressure and lipid profiles (27).

In line with a well-conducted meta-analysis showing no overall clinically significant benefit for low-carbohydrate diets (26), a systematic review and meta-analysis of 8 studies reporting remissions (n=264) found no benefit for T2D remissions at 6 or 12 months with low or very-low carbohydrate diets. There was a short-term advantage for achieving HbA1c<6.5% more often (57%) than control groups (31%) at 6 months. This effect was lost at 12 months (28).

The field investigating type 2 diabetes remission continues to grow, but at an overview of the current evidence points to remission being most likely after ‘double-digit’ weight loss (>10kg/%, preferably >15kg/%), achieved as early as possible following diagnosis. A TDR induction phase to initiate weight loss has the strongest evidence, with actively supported transition onto a sustainable eating pattern thereafter. Low carbohydrate diets may help a proportion of patients achieve sufficient weight loss, but further research and better long-term data are required for weight loss maintenance and lasting remissions before this can be routinely recommended.

## Remission in Real-Life

The success of DiRECT has led to improved service provision for the wider population of people living with T2D. For example, in the UK, a major investment was made by the Scottish Government to specifically target type 2 diabetes prevention and remission in all NHS health board areas, using the evidence-based DiRECT/Counterweight-Plus diet programme (29) NHS England is evaluating interventions for remission in 5,000 people with T2D (30), using a variety of approaches which follow the ‘DiRECT Principles’, with impressive preliminary results (31).

Although the DiRECT/Counterweight Plus programme is highly cost-effective, indeed cost-saving, current dietetic services are typically under-funded and under-staffed to manage the numbers of people with T2D, and as a result, access to this intervention is limited in comparison to the numbers eligible. Although funding is a major consideration, potential solutions to improve scalability include delivering TDR intervention in a group-setting, which also provides opportunity for peer support. This approach was used in the PREVIEW trial, which reported average weight loss of 11% body weight in over 2000 people with pre-diabetes taking part in an 8-week TDR group intervention, with very low drop out and only a fraction progressing to T2D at the 3-year study end point (32). Further evidence in support of group-based TDR programmes come from studies in people with osteoarthritis, sleep apnoea and psoriasis, where weight losses of 10-20% body weight have been documented (31, 33, 34).

Traditionally, weight management interventions have taken place in a face-to-face format. However, the healthcare delivery landscape has changed rapidly in recent years, with remotely delivered weight management programmes becoming commonplace due to restrictions imposed by Covid-19, together with significant advances in interactive digital technologies. Digital healthcare offers improved convenience and accessibility for many individuals, especially those of working age, and provides cost savings to health services. Importantly, outcomes for weight management appear to be at least comparable to traditional approaches (35). As healthcare delivery and diabetes care continues to evolve, and the demand for scalability to deal with the double burden of obesity and type 2 diabetes grows, it is likely that digital delivery will become the first line approach, and closer working partnerships between healthcare and industry will be required.

## Future Directions

The publicity around DiRECT and DIADEM-1 has raised public expectations, to the degree that a failure to achieve remission could be psychologically damaging, and demanding of management. For some, greater weight loss may be successful, but even after bariatric surgery, remission rates are similar to DiRECT. While most people diagnosed as T2D have a weight-loss-responsive disease characterised by ectopic fat accumulation in liver and pancreas, small numbers have a different cause of persistent beta-cell injury.

While losing weight is hard for people, in particular for those with longstanding obesity, maintaining weight loss is invariably a harder task, and specific research is needed to define how to optimise weight-loss maintenance. There is extensive and consistent RCT evidence that weight loss differences between diets are marginal, and optimising individual adherence appears to be more important than macronutrient composition (36) though tailoring diets to personal preference is not always associated with better outcomes (20). Managing life events, day-to-day stress and negative emotions is likely to be critical for weight loss maintenance in the long term (37). Regular physical activity and exercise on its own has only limited effect on weight loss for most people, but valuable for maintaining muscle mass and preventing weight regain (38). Modern weight management medications such as GLP-1 agonists operate using physiological pathways to control both appetite and insulin secretion after eating, and are much more potent, and safer, than older drugs. If combined with a good behavioural intervention, they are able to help many more people achieve and maintain weight losses of over 10%, or over 15% (39–41). These drugs are also glucose lowering so remission cannot be claimed without withdrawing them and implementing non-pharmacological weight-loss maintenance. Looking to the future, innovative approaches to prevent weight gain by stimulating endogenous GLP-1 and PYY are currently being trialed, using oral hydrogels (42) and food-products such as inulin propionate ester (43). Weight regain threatens the sustainability of T2D remission, and whilst weight loss maintenance is undermined by interacting physiological, behavioural and environmental factors, emerging pharmacotherapies used in combination with multi-component weight management programmes offer promise in addressing this problem.

## Conclusions

It is time to rethink treatment targets for T2D, and to aim for remission at diagnosis when motivation for change is often high, and disease progression is at an early stage. Despite expert guidelines and numerous medications to lower HbA1c, blood pressure and lipids, people diagnosed with T2D remain at risk of multiple complications over the long term, and substantial reductions in life expectancy. However, in light of evidence demonstrating that weight loss ≥10% body weight can reliably induce remission in most people early in the disease course, we can now offer people hope that a better prognosis is possible. This new knowledge is beginning to be translated into routine care settings and the adoption of digital technology by health care providers will facilitate future scalability and delivery of such interventions. Multi-component weight management incorporating an initial period of TDR offer the best bet for substantial weight loss and T2D remission, and with this in mind, we should no longer persist in offering people ineffective strategies for this treatment target, and instead prioritise programmes with a proven evidence base for delivering weight losses of ≥10% body weight.

## Data Availability Statement

The original contributions presented in the study are included in the article/supplementary material, further inquiries can be directed to the corresponding author.

## Author Contributions

JN drafted the manuscript. GT and ML critically reviewed the manuscript for important intellectual content, and edited drafts. All authors reviewed and approved the final manuscript.

## Funding

Aspects of this work were supported by the Toronto 3D Knowledge Synthesis and Clinical Trials foundation. This included journal publication costs, overheads costs, and author honoraria (JCN only).

## Conflict of Interest

JN has worked as a clinical research coordinator at INQUIS Clinical Research. He has also received research support from Glycemia Consulting Inc. GT reports funding for PhD fees, conference attendance and departmental research support from Cambridge Weight Plan outside the submitted work. ML has had departmental research funding from Diabetes UK, NIHR, Novo Nordisk, has received personal fees for lecturing and consultancy from Novo Nordisk, Merck, Eli Lilly, Sanofi, Nestle, Oviva, and has provided unpaid medical consultancy to Counterweight Ltd.

## Publisher’s Note

All claims expressed in this article are solely those of the authors and do not necessarily represent those of their affiliated organizations, or those of the publisher, the editors and the reviewers. Any product that may be evaluated in this article, or claim that may be made by its manufacturer, is not guaranteed or endorsed by the publisher.
